# Blood plasma lipidome profile of dairy cows during the transition period

**DOI:** 10.1186/s12917-015-0565-8

**Published:** 2015-10-07

**Authors:** S. Imhasly, C. Bieli, H. Naegeli, L. Nyström, M. Ruetten, C. Gerspach

**Affiliations:** Institute of Pharmacology and Toxicology, Vetsuisse Faculty, University of Zurich, Winterthurerstrasse 260, 8057 Zurich, Switzerland; Department of Farm Animals, Vetsuisse Faculty, University of Zurich, Winterthurerstrasse 260, 8057 Zurich, Switzerland; Institute of Food, Nutrition and Health, Swiss Federal Institute of Technology, Schmelzbergstrasse 9, 8092 Zurich, Switzerland; Institute of Veterinary Pathology, Vetsuisse Faculty, University of Zurich, Winterthurerstrasse 260, Zurich, Switzerland

## Abstract

**Background:**

The transition period of dairy cows, around parturition and the onset of lactation, involves endocrine and metabolic changes to compensate for an increased energy requirement aggravated by reduced feed intake. Transition cows adjust to the resulting negative energy balance with the mobilization of lipids from the adipose tissues yielding increased blood levels of non-esterified fatty acids and ketone bodies like *β*-hydroxybutyrate.

**Results:**

To study the biochemical adaptations underlying this physiologic adjustment and possible pathologic derangements, we analyzed the blood plasma lipidome of transition cows by ultra-pressure liquid chromatography coupled to high-resolution quadrupole time-of-flight mass spectrometry. The resulting data were processed by principal component analysis, revealing over 60 lipid masses that change in abundance over the test period ranging from two weeks before calving to four weeks postpartum. Further characterization of analytes by tandem mass spectrometry demonstrated that the concentration of triacylglycerides in plasma drops at the day of parturition whereas the plasma level of many phosphatidylcholines and two sphingomyelins increases steadily during early lactation.

**Conclusion:**

This newly identified shift in phospholipid composition delivers a potential biomarker to detect aberrant metabolic pathways in transition cows and also provides insights into how to prevent and treat associated disorders like fatty liver disease.

## Background

During the last few weeks before parturition and the first weeks post partum, dairy cows have to undergo the physiologic transition from pregnancy to lactation, which involves many endocrine and metabolic adaptations related to parturition and the onset of milk production (Grummer et al. 1995; Huzzey et al. 2005). A key problem during this transition period is the dramatic increase in energy requirements for milk production, paralleled by a decreased feed intake, occurring around parturition and the first week after calving (Moyes et al. 2013). This condition results in a negative energy balance, to which dairy cows adjust by lipid mobilization from the adipose tissue representing a major fuel source during the transition period (Sordillo and Raphael 2013). However, lipid mobilization leads to an increase in non-esterified fatty acid (NEFA) concentrations, which are linked to greater incidences of ketosis, displaced abomasum and retained placenta (Dyk 1995). Many transition period disorders, including fatty liver, also occur in a subclinical form affecting the overall health status, milk production and reproductive performance of dairy cows.

Fatty liver is a multifactorial metabolic disorder resulting from an increased flux of NEFAs to the liver, followed by their reconversion to triacylglycerides and storage as intracellular lipid droplets (Bobe et al. 2004). The development of fatty liver disease in cows with a negative energy balance depends on the level of overall lipid mobilization, the rate of fatty acid oxidation in the liver and the efficiency of fatty acid elimination from the liver, either as ketone bodies reflecting the fatty acid breakdown or as full triacylglycerides exported in the form of very low density lipoproteins (VLDLs) (Ingvartsen 2006). Fatty liver is related to other diseases including retained placenta, uterine infection, milk fever, abomasal displacement and mastitis. These pathological changes affect up to 50 % of dairy cows during early lactation (Sejersen et al. 2012; Jorritsma et al. 2001).

A liver biopsy remains the only diagnostic tool to determine the lipid content of the liver and, as a consequence, the extent of the disease is in practice frequently uncertain. A non-invasive and accurate test for the diagnosis of fatty liver disease would, therefore, be helpful for the management of this disorder in individual animals as well as on a herd level. Such a simplified test would also allow to investigate the incidence and risk factors of the disorder (Ingvartsen 2006). Because the lipid metabolism is a key aspect of the physiology of transition cows (Drackley 1999; Gross et al. 2013), we conducted a comprehensive analysis of the plasma lipidome of cattle during the time before and after parturition. This study reveals that the physiologic adaptation to an increased energy requirement of transition cows involves a post partum elevation of phosphatidylcholines and sphingomyelins in their blood plasma.

## Methods

### Animals and management

Twelve dairy cows of the Holstein Frisian breed were included in this study. In order to reduce variability, all cows were selected from the same farm. The animals were housed in an open spaced barn comprising a total of 75 dairy cows. To be included in the study, the cows had to be multiparous, clinically healthy and not receiving any treatments at the beginning of the study. Also, they had to be confirmed pregnant with a known breeding date. Their health status was assessed by the herd veterinarian and clinicians of the Department of Farm Animals. All cows of the study were fed exactly the same diet and kept in the same barn. During the preceding dry period, the cows were fed grass silage, straw, hay and a mineral supplement. Two weeks before parturition, the cows were regrouped to the lactating animals receiving increasing amounts of a concentrate (0–1.5 kg). Post partum, the cows were fed grass and corn silage, beet pulp, soybean, grass, mineral supplement, and up to 4 kg concentrate. The body condition score was recorded concurrently with each blood sample collection.

The study was approved by the Veterinary Office of the Kanton Zurich and conducted in accordance with guidelines established by the Animal Welfare Act of Switzerland (permission No. 27/2013). Consent was obtained from the owner of the dairy farm to collect samples from his cows for the current study.

### Collection of blood samples and liver tissue

Samples were obtained from the jugular vein at days −14, −7, 0, +7, +14, +21 and +28 relative to parturition. The samples were drawn always at the same time in the afternoon. Tubes supplemented with lithium heparin (Sarstedt AG and Co., Nümbrecht, Germany) were used to collect 10 ml of blood. The heparinized blood was centrifuged at 4000 g for 5 min. Separated plasma was stored in 2-ml tubes at −80 °C until analysis. Plasma samples were used for lipidome evaluation and measurement of -hydroxybutyrate and NEFA.

Liver biopsies were taken as described (Mølgaard et al. 2012) under ultrasonographic control and stained with hematoxylin-eosin (H & E). The histological lesions were staged into four categories: no abnormalities, mild fatty liver (only cells from one liver zone affected by lipid vacuoles), moderate fatty liver (fatty degeneration of the periportal to midzonal or midzonal to centrolobular areas), severe fatty liver (all three zones affected including the Kupffer cells).

### Measurement of NEFA and *β*-hydroxybutyrate

Plasma concentrations of NEFA and *β*-hydroxybutyrate were determined by enzymatic analyses using the Wako NEFA-HR (2) (Wako Chemicals GmbH, Neuss, Germany) and the *β*-Hydroxybutyrate LiquiColor® kit (Stanbio Laboratory, Boerne, TX, USA), respectively. Spectrophotometric measurements were performed for both NEFA and *β*-hydroxybutyrate, using a Cobas Mira S Chemistry Analyzer (Roche, Basel, Switzerland).

### Lipid nomenclature

For the designation of lipids, the common standard lipid language described by the Lipidomics Gateway (http://www.lipidmaps.org, National Institute of General Medical Sciences, National Institutes of Health) and by Schmelzer et al. (2007) was applied.

### Chemicals and internal standards

All solvents were liquid chromatography-grade. Leucine-enkephalin was used as the lock mass at a concentration of 1 ng/μl in a solution of acetonitrile/water (50:50, v/v) supplemented with 0.1 % formic acid.

For the internal standards, an exogenous mixture of lipids was added to the organic solvent during the initial extraction step. These standards consisted of 1-heptadecanoyl-2-hydroxy-*sn*-glycero-3-phosphocholine (LPC 17:0/0:0), 1-nonadecanoyl-2-hydroxy-sn-glycero-3-phosphocholine (LPC 19:0/0:0), 1,2-dipentadecanoyl-*sn*-glycero-3-phosphoethanolamine (PE 15:0/15:0), 1,2-diheptade-canoyl-*sn*-glycero-3-phosphoethanolamine (PE 17:0/17:0), 1,2-dipentadecanoyl-*sn*-glycero-3-phospho-(1’-*rac*-glycerol) sodium salt (PG 15:0/15:0), 1,2-diheptadecanoyl-*sn*-glycero-3-phospho-*rac*-(1-glycerol) sodium salt (PG 17:0/17:0), 1,2-ditridecanoyl-*sn*-glycero-3-phosphocholine (PC 13:0/13:0), 1,2-ditricosanoyl-*sn*-glycero-3-phosphocholine (PC 23:0/23:0), 1,2-dinona-decanoyl-*sn*-glycero-3-phosphocholine (PC 19:0/19:0) and 1,2-di-(3,7,11,15-tetramethylhexadecanoyl)-*sn*-glycero-3-phosphocholine [PC 16:0 (3me,7me,11me,15me)/16:0 (3me,7me,11me,15me)] purchased from Avanti Polar Lipids (Alabaster, AL, USA); 1,2,3-tripentadecanoylglycerol (TG 15:0/15:0/15:0) and 1,2,3-trihepta-decanoylglycerol (TG 17:0/17:0/17:0) were from Sigma-Aldrich (Buchs, Switzerland). The concentration of internal standards (350 nM) was calculated relative to the final amount of organic solvent in the extraction tube.

### Sample extraction

Lipids were extracted from bovine plasma following a published protocol (Matyash et al. 2008). The extraction was validated by the addition of internal standards. For that purpose, 127 μl methanol was placed into 1.5-ml Eppendorf tubes and 23 μl of the 10 μM internal standard mixture was added (final concentration of 350 nM in the organic phase). A sample aliquot of 20 μl was added to each tube and the mixtures vortexed for 10 s. The mixtures were supplemented with 500 μl of methyl-*tert*-butyl ether and incubated for 60 min at room temperature in a thermo shaker (Vaudaux-Eppendorf AG, Schönenbuch, Basel) at 800 rpm. Then, a phase separation was induced by adding 125 μl of water and further vortexing. The samples were centrifuged for 10 min at 1000 g and 4 °C; 300 μl of the upper (organic) phase containing the lipids and non-polar components were collected and transferred into a new tube. The organic phases were dried in a vacuum centrifuge (Savant Speed Vac Plus SC 110A, Savant Instruments Inc., Holbrook, NY, USA), dissolved in 300 μl methanol and stored at −80 °C until measurements.

### Liquid chromatography

An Acquity UPLC (Waters, Milford, MA, USA) system was used for ultra-pressure chromatographic sample separations. The plasma extracts were injected as triplicates and in a random order onto a HSS T3 column (Waters, Milford, MA, 1.8-μm particle, 100 × 2.1 mm id) heated to 55 °C. The average column pressure was 7000 psi. A binary gradient of two solvent mixtures was used for elution. Eluent A consisted of acetonitrile and water (50:40, v/v) with 10 mM ammonium acetate; eluent B consisted of acetonitrile and isopropanol (10:90, v/v) with 10 mM ammonium acetate. Eluent A was used for weak needle washes, whereas isopropanol was used for strong needle washes. The flow rate was 0.4 ml/min and the injection volume 10 μl. A linear gradient was performed for the sample analysis. The initial portion of the gradient was held at 60 % A and 40 % B. In the next 10 min, the composition was changed in a linearly ramped gradient (curve 6)–100 % B and held for 2 min. The system was switched back to the initial proportion (60 % A, 40 % B) and the column was equilibrated for 3 min. The total run time was 15 min.

### Mass spectrometry

The UPLC inlet was coupled to a quadrupole time-of-flight mass spectrometer (SYNAPT G2 HDMS, Waters, MS Technologies Manchester, U.K.). Mass spectrometry was carried out following the protocol of Castro-Perez and Kamphorst (2010), whereby electrospray ionization (ESI) was employed in the positive and negative mode. A capillary voltage of 3 kV and cone voltage of 30 V were used for both polarities. The desolvation source conditions involved desolvation gas at 700 L/h and a temperature of 400 °C. The mass range during acquisition was 50–1200 Da and the signals were acquired in the centroid mode. Leucine-enkephalin was used as internal reference in each measurement.

During data acquisition, the first quadrupole was operated in a wide band RF mode, such that all ions were able to enter the T-wave collision cell. In this cell, two acquisition functions were applied. The first function with 5 eV resulted in non-fragmented ion molecules while the second function used a collision energy ramp of 20–30 eV to generate fragmented ions (MS^E^ method). Argon gas was used for collision-induced dissociation. Using this interleaved acquisition, fragmented and non-fragmented ions could be used for quantification and initial ion identification. For final ion identification, a tandem mass spectrometry (MS/MS) method was applied by setting the energy ramp for collision-induced dissociation at 15–40 eV. The fragmentation pattern resulting from each parental mass was identified using the mass spectrometry database provided online the Lipidomics Gateway (http://www.lipidmaps.org, National Institute of General Medical Sciences, National Institutes of Health).

### Data analysis

Descriptive statistics of the concentrations of NEFA and -hydroxybutyrate was performed using graphpad GraphPad Prism 6 (Graph Pad Software, La Jolla, CA, USA). The open source program MZmine (http://mzmine.sourceforge.net; Project GNU free software foundation, Boston, USA) was used for automatic alignment, denoising, deconvolution and extraction of peaks (Pluskal et al. 2010). All data were standardized using exogenous standards. The follow-up statistics were performed with the open source program R (R_Core_Team R Foundation for Statistical Computing 2014) and GraphPad Prism 6.

## Results

**Fig. 1:**
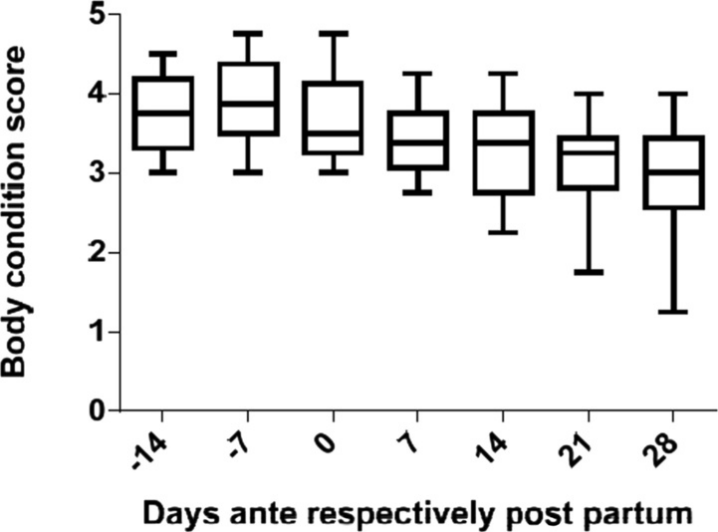
Clinical examination. History of the body condition score of the 12 cows over the experimental period (*median values with range*). The time point of clinical examination is indicated relative to the date of calving

A total of 12 multiparous cows of the Holstein Frisian breed, kept in the same farm, were tested for changes in the blood plasma lipidome occurring during the peripartal transition phase. The selected cows were aged between 3 and 12 years (mean age: 5 ± 2.6 years) and undergoing their 2^nd^ to 10^th^ lactation (mean number of lactations 3 ± 2.3). The mean dry period lasted 64 ± 14.27 days. The animals were clinically healthy at the beginning of the study, but the histologic findings by means of liver biopsies taken four weeks after calving showed that only one animal remained completely devoid of fatty degeneration of hepatocytes; 7 cows displayed a mild and 4 cows a moderate degree of lipid deposition in hepatocytes. Importantly, however, none of these animals developed clinical symptoms of fatty liver disease although several cows experienced other health problems after parturition including hypocalcemia (*n* = 7), retained placenta with subsequent metritis (*n* = 2), mastitis (*n* = 1), and lameness (*n* = 4). Accordingly, the overall body condition score of the 12 cows deteriorated slightly with progression of the transition period (Fig. 1).

### Clinical chemistry analysis

**Fig. 2:**
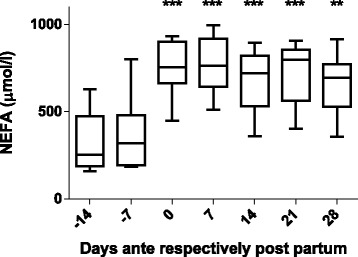
NEFA levels. History of NEFA concentrations in the plasma of the 12 cows over the experimental period (*median values with range*). The time points are indicated relative to the date of calving. ***P < 0.001 compared to NEFA concentrations at the beginning of the study (day −14). ANOVA: F (6,77) = 14.74, *P* value < 0.0001

**Fig. 3:**
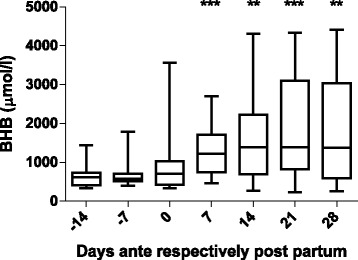
Ketone bodies. History of *β*-hydroxybutyrate (BHB) concentrations in the plasma of the 12 cows over the experimental period (*median values with range*). The time points are indicated relative to the date of calving. **P < 0.01 and ***P < 0.001 compared to *β*-hydroxybutyrate concentrations at the beginning of the study (day −14). ANOVA F (6,77) = 3.333, *P* value = 0.0057

Blood samples were obtained ante partum (on days −14 and −7) on the day of calving and post partum (on days +7, +14, +21 and +28). The blood plasma concentration of NEFAs increased from low values before calving (for example 360 ± 190 μmol/l on day −7) to significantly higher values post partum, reaching a maximum at day +7 (770 ± 160 μmol/l). These NEFA concentrations remained at high levels until at least day +28 (Fig. 2). The plasma concentrations of -hydroxybutyrate displayed some variability among the individual cows. However, a trend of increasing -hydroxybutyrate levels as the transition period progressed could be detected with significantly higher concentrations in the plasma post partum (for example 1814 ± 1407 μmol/l on day +28) compared to the samples taken ante partum (for example 632 ± 299 μmol/l on day −14) (Fig. 3).

### Plasma lipidome analysis

**Fig. 4:**
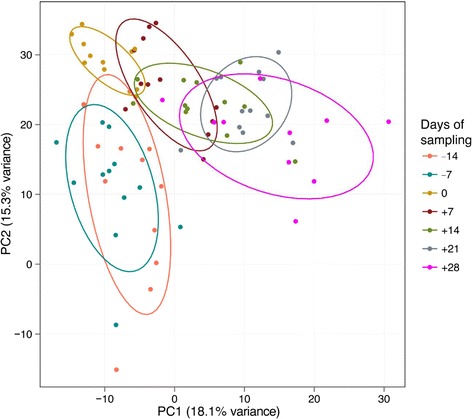
Principal component analysis. Principal components were determined with all 579 detected masses of the 82 measured samples and arranged in 7 groups according to the time of sampling relative to the date of calving (−14 days to +28 days)

The lipid composition of plasma samples obtained at the different time points were compared by liquid chromatography coupled to quadrupole time-of-flight mass spectrometry. This analysis yielded over 500 masses detected in the positive ion mode, and over 200 masses in the negative ion mode, representing lipids and other non-polar metabolites. To illustrate the differences in the lipidome profile at different stages of the transition period, from 14 days ante partum to 28 days post partum, the multivariate data were subjected to principal component analysis (PCA), which is an unsupervised clustering method that provides an overview of the results by reducing the dimensionality of complex findings. The principal components of the data acquired in the positive ion mode were graphically plotted as a score plot (Fig. 4), thus revealing that the samples from each time point cluster together forming distinguishable groups. Instead, the masses acquired in the negative ion mode did not shown any such clustering (data not shown).

**Fig. 5:**
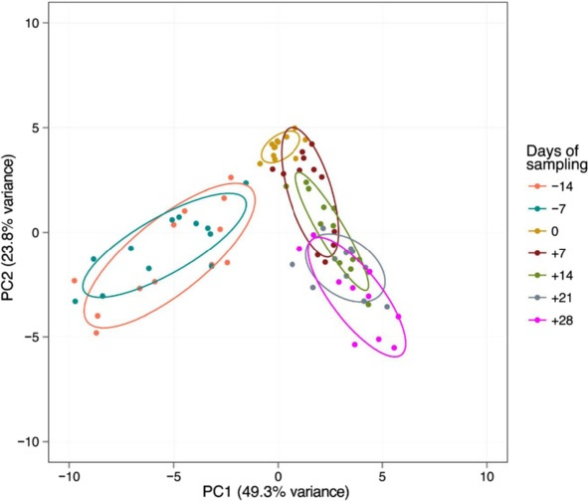
Principal component analysis. Principal components were re-calculated focusing on the 32 identified masses and arranged in 7 groups according to the time of sampling relative to the date of calving (−14 days to +28 days)

**Fig. 6:**
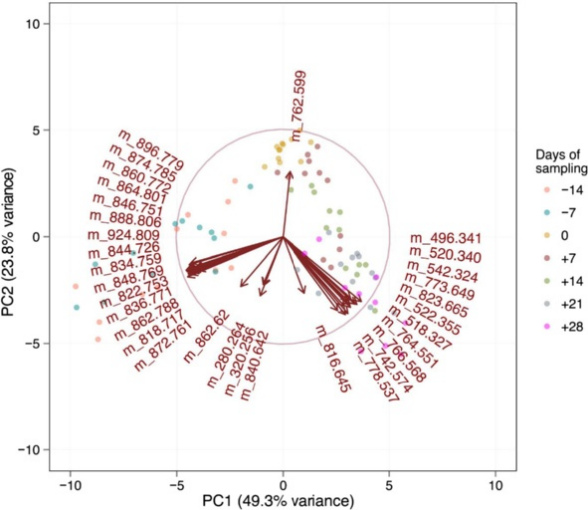
Plot of the variable factors. Values were extracted from the principal component analysis obtained with 32 identified masses. The time of sampling is indicated relative to the date of calving (−14 days to +28 days)

The data of 40 masses that best correlate with the principal component 1 (PC1), and collections of 20 masses that each correlate best with the principal components 2 and 3 (PC2 and PC3) were extracted. These 80 masses accounted for all the observed separation between the seven time points in the PCA score plot. Due to duplicate representations of the same masses in the different PCs, finally 62 masses were selected for further investigation. From these, 32 masses could be identified by tandem mass spectrometry (Table 1). The score plot was then replicated using the data of these 32 identified masses, yielding a closer clustering of the samples obtained from individual time points (Fig. 5). An additional vector plot of the variables evidenced the particular contribution of each of the 32 identified masses to the overall separation between the different time points around parturition (Fig. 6).

**Table 1:** Identified masses by tandem mass spectrometry

Class	Formula	m/z	Annotation	Lonization mode
lysoPC	C_24_H_50_NO_7_P	496.341	LPC 16:0	[M + H]^+^
	C_26_H_50_NO_7_P	520.340	LPC 18:2	[M + H]^+^
	C_26_H_52_NO_7_P	522.355	LPC 18:1	[M + H]^+^
	C_28_H_48_NO_7_P	542.324	LPC 20:5	[M + H]^+^
	C_26_H_48_NO_7_P	518.324	LPC 18:3	[M + H]^+^
PC	C_42_H_80_NO_7_P	742.574	PC P-34:2	[M + H]^+^
	C_42_H_84_NO_8_P	762.599	PC 34:0	[M + H]^+^
	C_44_H_78_NO_7_P	764.551	PC P-36:5	[M + H]^+^
	C_44_H_80_NO_7_P	766.568	PC P-36:4	[M + H]^+^
	C_44_H_76_NO_8_P	778.537	PC 36:6	[M + H]^+^
	C_46_H_90_NO_8_P	816.645	PC 38:1	[M + H]^+^
	C_48_H_90_NO_8_P	840.642	PC 40:3	[M + H]^+^
	C_50_H_88_NO_8_P	862.620	PC 42:6	[M + H]^+^
SM	C_48_H_91_N_2_O_6_P	773.649	SM 39:1	[M + H]^+^
	C_48_H_91_N_2_O_6_P	823.665	SM 43:3	[M + H]^+^
Fatty amides	C_18_H_33_NO	280.264	Linoleamide	[M + H]^+^
	C_20_H_33_NO_2_	320.256	Anandamide	[M + H]^+^
TG	C_51_H_92_O_6_	818.717	TG 48:3	[M + NH4]^+^
	C_51_H_96_O_6_	822.753	TG 48:1	[M + NH4]^+^
	C_52_H_96_O_6_	834.759	TG 49:2	[M + NH4]^+^
	C_52_H_98_O_6_	836.771	TG 49:1	[M + NH4]^+^
	C_53_H_94_O_6_	844.726	TG 50:4	[M + NH4]^+^
	C_53_H_96_O_6_	846.751	TG 50:3	[M + NH4]^+^
	C_53_H_98_O_6_	848.769	TG 50:2	[M + NH4]^+^
	C_54_H_98_O_6_	860.772	TG 51:3	[M + NH4]^+^
	C_54_H_100_O_6_	862.788	TG 51:2	[M + NH4]^+^
	C_54_H_102_O_6_	864.801	TG 51:1	[M + NH4]^+^
	C_55_H_98_O_6_	872.761	TG 52:4	[M + NH4]^+^
	C_55_H_100_O_6_	874.785	TG 52:3	[M + NH4]^+^
	C_56_H_102_O_6_	888.806	TG 53:3	[M + NH4]^+^
	C_57_H_98_O_6_	896.779	TG 54:6	[M + NH4]^+^
	C_59_H_102_O_6_	924.809	TG 56:6	[M + NH4]^+^

**Fig. 7:**
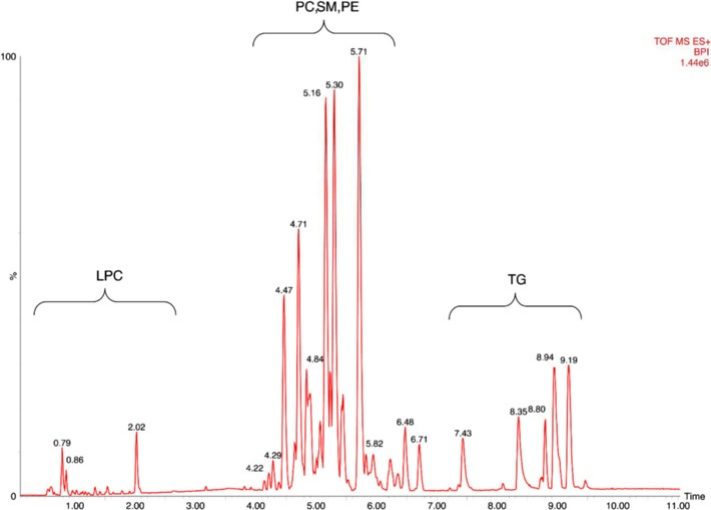
Ultra-pressure liquid chromatogram. This chromatographic profile of a representative sample demonstrates the distinct elution windows of different lipid classes identified by mass spectrometry. LPC, lyso-phosphatidylcholines; PC, phosphatidylcholines; SM, sphyngomyelins; PE, phosphoethanolamines; TG, triacylglycerides

**Fig. 8:**
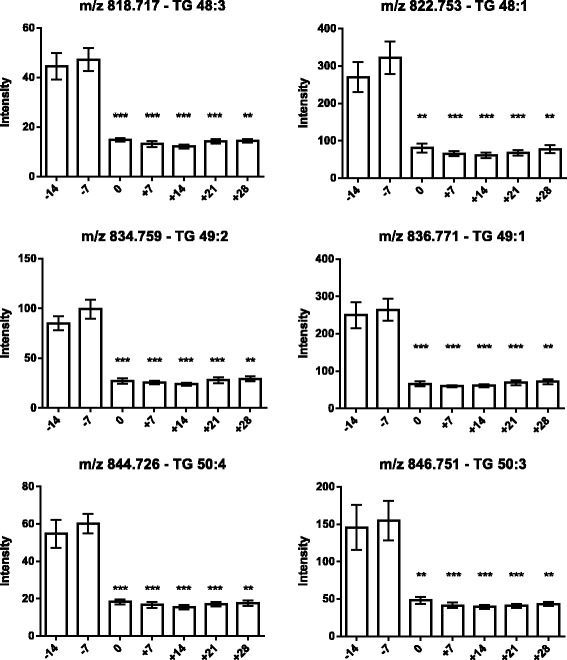
Plasma concentration of triacylglycerides (TG 48:3, TG 48:1, TG 49:2, TG 49:1, TG 50:4 and TG 50:3) in the 12 transition period cows. The time of sampling is indicated relative to the date of calving. Mean values ± SEM; **P < 0.01 and ***P < 0.001 compared to triacylglyceride concentrations at the beginning of the study (day −14)

**Fig. 9:**
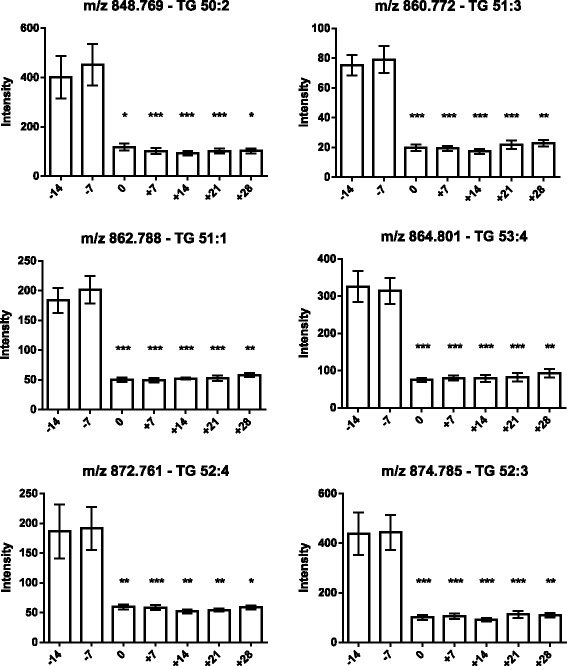
Plasma concentration of triacylglycerides (TG 50:2, TG 51:3, TG 51:1, TG 53:4, TG 52:4 and TG 52:3) in the 12 transition period cows. The time of sampling is indicated relative to the date of calving. Mean values ± SEM; *P < 0.05, **P < 0.01 and ***P < 0.001 compared to the values at the beginning of the study (day −14)

**Fig. 10:**
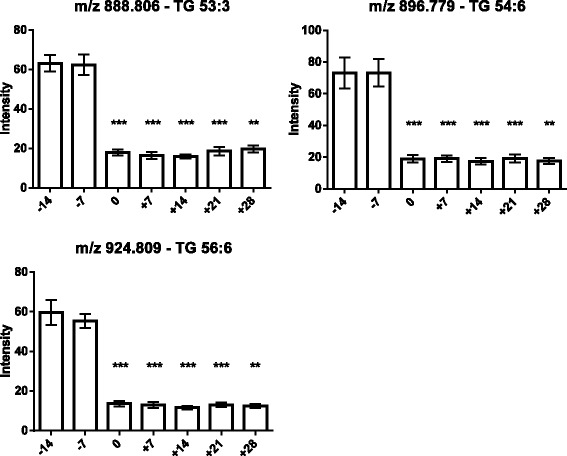
Plasma concentration of triacylglycerides (TG 53:3, TG 54:6 and TG 56:6) in the 12 transition period cows. The time of sampling is indicated relative to the date of calving. Mean values ± SEM; *P < 0.05, **P < 0.01 and ***P < 0.001 compared to the values at the beginning of the study (day −14)

**Fig. 11:**
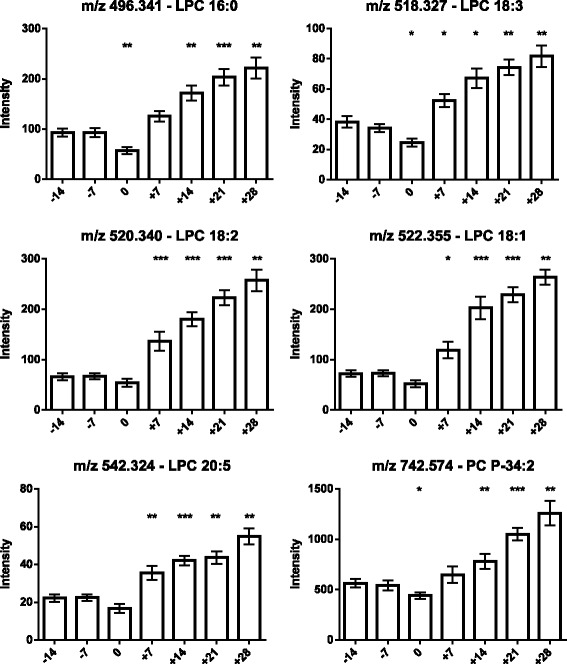
Plasma concentration of lyso-phosphatidylcholines (LPC 16:0, LPC 18:3, LPC 18:2, LPC 18:1 and LPC 20:5) and phosphatidylcholines (PC P-34:2) in the 12 transition period cows. The time of sampling is indicated relative to the date of calving. Mean values ± SEM; *P < 0.05, **P < 0.01 and ***P < 0.001 compared to the values at the beginning of the study (day −14)

**Fig. 12:**
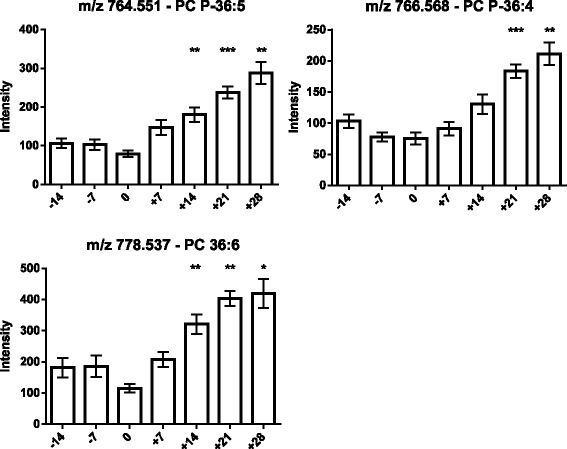
Plasma concentration of phosphatidylcholines (PC P-36:5, PC P-36.4 and PC 36:6) in the 12 transition period cows. The time of sampling is indicated relative to the date of calving. Mean values ± SEM; *P < 0.05, **P < 0.01 and ***P < 0.001 compared to the values at the beginning of the study (day −14)

**Fig. 13:**
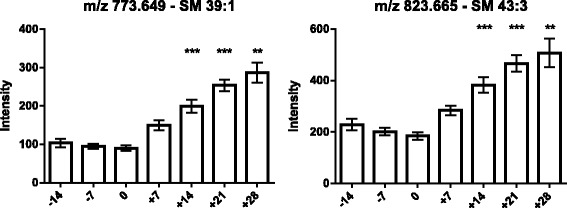
Plasma concentration of sphingomyelines (SM 39:1 and SM 43:3) in the 12 transition period cows. The time of sampling is indicated relative to the date of calving. Mean values ± SEM; **P < 0.01 and ***P < 0.001 compared to the values at the beginning of the study (day −14)

**Fig. 14:**
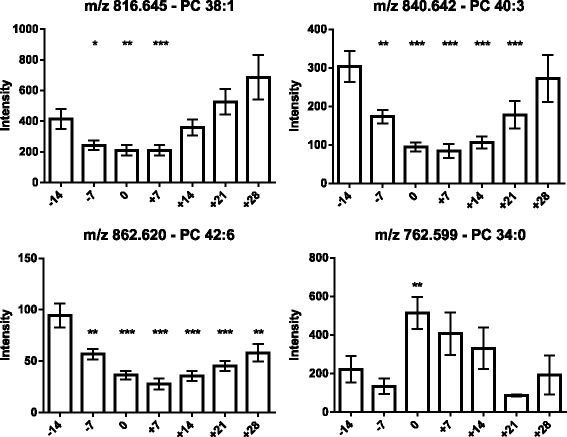
Plasma concentration of phosphatidylcholines (PC 38:1, PC 40:3, PC 42:6 and PC 34:0) in the 12 transition period cows. The time of sampling is indicated relative to the date of calving. Mean values ± SEM; *P < 0.05, **P < 0.01 and ***P < 0.001 compared to the values at the beginning of the study (day −14)

**Fig. 15:**
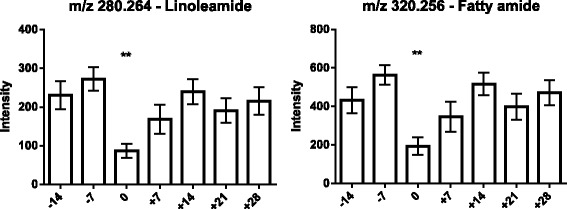
Plasma concentration of fatty acid amides in the 12 transition period cows. The time of sampling is indicated relative to the date of calving. Mean values ± SEM; **P < 0.01 and ***P < 0.001 compared to the values at the beginning of the study (day −14)

As the next step, the lipids responsible for this separation were characterized and quantified. In Fig. 7, the chromatographic retention time windows of lipid classes are indicated to illustrate how their classification is simplified by the respective position in the elution profile (Ogiso et al. 2008). One class of lipids that discriminates between the different time points around parturition consisted of triacylglycerides (TGs), including TG 48:3, TG 48:1, TG 49:2, TG 49:1, TG 50:4, TG 50:3, TG 50:2, TG 51:3, TG 51:2, TG 51:1, TG 52:4, TG 52:3, TG 53:3, TG 54:6 and TG 56:6. All these TGs displayed high plasma levels in cows before calving but, on the day of parturition, their levels dropped instantaneously and remained low for up to 28 days post partum (Figs. 8, 9 and 10). Lyso-posphatidylcholine (LPC) and phosphatidylcholine (PC) levels, in contrast, increased progressively post partum. This increment was significant for LPC 16:0, LPC 18:3, LPC 18:2, LPC 18:1, LPC 20:5, PC P-34:2, PC P-36:5, PC P-36:4 and PC 36:6 (Figs 11 and 12). In addition, the sphingomyelines SM 39:1 and 43:3 were increased post partum in the same manner as described for phosphatidylcholines (Fig. 13). There is an intriguing although not statistically significant drop of PC levels at the day of parturition. It should be noted that not all identified phosphatidylcholines showed this same pattern of increased concentrations post partum compared to ante partum. In the case PC 38:1, PC 40:3 and PC 42:6, there was first a drop in plasma levels from day −14 to calving, followed by a slow recovery post partum (Fig. 14). For PC 34:0, we observed an exceptionally sharp increase at the day of calving followed by a progressive reduction to reach the starting level measured at the beginning of the study (Fig. 14). Finally, we also identified the two fatty acid amides linoleamide and anandamide, whose plasma levels are transiently depressed only at the time of calving (Fig. 15).

## Discussion

During the transition from pregnancy to lactation, dairy cows experience a challenging period with increasing energy requirements for maintenance, fetal growth in late pregnancy, mammary tissue remodeling and early lactation (Herdt 2000; Esposito et al. 2014). Despite the enhanced nutritional demands, this period coincides with a reduction in dry matter intake (Ingvartsen and Andersen 2000). The resulting negative energy balance is considered a normal occurrence in dairy cows after calving (McArt et al. 2013), but only animals poorly adapting to these changing physiologic conditions are at risk of developing various production disorders.

Several potential biomarkers have already been evaluated for their ability to predict which cows are at risk of diseases related to the transition period. Most tested parameters reflect the negative energy balance and the rate of hepatic fatty acid oxidation, which can be demonstrated by measuring NEFAs and *β*-hydroxybutyrate, respectively. Increased concentrations of these metabolites ante partum have been associated with higher odds of displaced abomasum, poor lactation and reduced fertility (Chapinal et al. 2012; Ospina et al. 2010). However, there is an urgent need for a non-invasive and accurate test to diagnose early alterations in the lipid metabolism leading to fatty liver disease. For that specific purpose, we used a large-scale and untargeted metabolomic approach, based on high-resolution quadrupole time-of-flight mass spectrometry, to analyze the physiologic lipidome of healthy dairy cows. Since metabolic profiles begin to change by the end of gestation (Holtenius et al. 2003; Bossaert et al. 2008), this investigation focused on a period of 6 weeks around parturition. There was no overt effect of age on lipid metabolism across the 12 cows of this study.

As expected (Ospina, P a et al. 2010; McArt, Jessica a a et al. 2013), the tested animals responded to their negative energy balance by mobilizing large amounts of body fat, leading to increased NEFA concentrations in plasma starting from the day of calving (Fig. 2). Similarly, −hydroxybutyrate levels in plasma increased post partum (Fig. 3) as a marker of lipid catabolism. In parallel, the overall lipidome profile changed abruptly on the day of calving with further intriguing adaptations post partum (Fig. 5). Our large-scale approach demonstrates that two main lipid classes undergo significant shifts in their plasma concentration during the transition period of dairy cows, i.e., triacylglycerides and phospholipids. The 15 unequivocally identified triacylglycerides were all characterized by a sharply reduced plasma concentration on the day of calving and post partum compared to ante partum values (see for example Fig. 8). This observation reflects the suddenly enhanced energy requirement and is in agreement with previous studies (Van den Top et al. 2005; Kessler et al. 2014). In contrast, the plasma concentration of the vast majority of the identified phospholipids (five lyso-phosphatidylcholines, four phosphathidylcholines and two sphingomyelins) increased steadily with progression of early lactation post partum (see for example Fig. 12). A common feature of these phospholipids is their requirement for the secretion of hepatic triacylglycerides as VLDL particles, indicating that a reduced level of phosphatidylcholines and other phospholipids may directly cause an excessive accumulation of triacylglycerides in the liver (Artegoitia et al. 2014; Côté et al. 2014). The transient drop of phosphatidylcholine levels at the day of parturition may be due to a sudden increase of phospholipase activity in order to gain energy from PCs (Jacobs et al. 2013). In addition to insufficient hepatic VLDL secretion, the drop in triacylglycerides and concomitant rise of phosphatidylcholines around parturitation can be due to an increase in mammary lipoprotein lipase activity as reported by Van den Top et al. (2005).

Further studies are now required to confirm a causal relationship between reduced phospholipid concentrations and fatty liver disease in dairy cows. On that line, we recently reported that dairy cows affected by fatty liver disease during the transition period display lower levels of phosphatidylcholines and sphingomyelins in their blood plasma than control animals (Imhasly et al. 2014). Thus, the abnormal decline of certain specific phosphatidylcholines and sphingomyelins could be regarded as a promising biomarker indicative of fatty liver disease. Conversely, dietary supplementation of biochemical precursors of phosphatidylcholines and sphingomyelins may help to prevent the occurrence of fatty liver disease in dairy cows. Additionally, it should be tested if direct intravenous infusion of missing phosphatidylcholines and sphingomyelins may serve as a new therapeutic strategy for the management of animals affected by severe-grade fatty liver disease.

## Conclusion

In the present study, a new lipidomic approach was used to demonstrate adaptations in the lipid metabolism to high energy demands, during the transition period. With this approach, using multivariate statistics and tandem mass spectrometry, we identified 32 masses, discriminating between time points, during the transition period. Most intriguing was the alteration in phosphatidylcholines, which can be linked to altered VLDL formation, which is a key factor in the pathogenesis of fatty liver disease. This newly identified shift in phospholipid composition delivers a potential biomarker to detect aberrant metabolic pathways in transition cows and provides insights into how to prevent and treat associated disorders. Future studies with larger animal groups will be needed, to validate the potential new biomarkers.

## Abbreviations

NEFA: Non-esterified fatty acid
VLDL: Very low density lipoprotein
MTBE: Methyl-*tert* butyl ether
UPLC: Ultra-performance liquid chromatography
ESI: Electrospray ionization
MS/MS: Tandem mass spectrometry
PCA: Principal component analysis
TG: Triacylglycerid
LPC: Lyso-phosphatidylcholine
PC: phosphatidylcholine

## References

[CR1] Artegoitia VM, Virginia M, Middleton JL, Harte FM, Campagna SR, de Veth MJ (2014). Choline and Choline Metabolite Patterns and Associations in Blood and Milk during Lactation in Dairy Cows. PLoS One.

[CR2] Bobe G, Young JW, Beitz DC (2004). Invited review: pathology, etiology, prevention, and treatment of fatty liver in dairy cows. J Dairy Sci.

[CR3] Bossaert P, Leroy JL, De Vliegher S, Opsomer G. 2008. “Interrelations between Glucose-Induced Insulin Response, Metabolic Indicators, and Time of First Ovulation in High-Yielding Dairy Cows.” J Dairy Sci 91(9). Elsevier: 3363–71. doi:10.3168/jds.2008-0994. 10.3168/jds.2008-0994 18765595

[CR4] Castro-Perez JM, Kamphorst J. 2010. “Comprehensive LC− MSE Lipidomic Analysis Using a Shotgun Approach and Its Application to Biomarker Detection and Identification in Osteoarthritis Patients.” J. Proteome Res. 9 (5): 2377–89. http://pubs.acs.org/doi/abs/10.1021/pr901094j. 10.1021/pr901094j 20355720

[CR5] Chapinal N, Carson ME, LeBlanc SJ, Leslie KE, Godden S, Capel M, Santos JE, Overton MW, Duffield TF. 2012. “The Association of Serum Metabolites in the Transition Period with Milk Production and Early-Lactation Reproductive Performance.” J Dairy Sci95(3). Elsevier: 1301–9. doi:10.3168/jds.2011-4724. 10.3168/jds.2011-4724 22365212

[CR6] Côté I, Chapados N, Jean-Marc L (2014). “Impaired VLDL Assembly: A Novel Mechanism Contributing to Hepatic Lipid Accumulation Following Ovariectomy and High-Fat/high-Cholesterol Diets?”. Br J Nutr.

[CR7] Drackley JK. 1999. “Biology of Dairy Cows During the Transition Period: The Final Frontier?” J Dairy Sci 82(11). Elsevier: 2259–73. doi:10.3168/jds.S0022-0302(99)75474-3. 10.3168/jds.s0022-0302(99)75474-3 10575597

[CR8] Dyk PB. 1995. “The Association of Prepartum Non-Esterified Fatty Acids and Body Condition with Peripartum Health Problems on 95 Michigan Dairy Farms.” Cincinnati, Ohio: Michigan State University.

[CR9] Esposito G, Irons PC, Webb EC, Chapwanya A. 2014. “Interactions between Negative Energy Balance, Metabolic Diseases, Uterine Health and Immune Response in Transition Dairy Cows.” Anim Reprod Sci 144(3–4). Elsevier B.V.: 60–71. doi:10.1016/j.anireprosci.2013.11.007. 10.1016/j.anireprosci.2013.11.007 24378117

[CR10] Gross JJ, Schwarz FJ, Eder K, van Dorland HA, Bruckmaier RM. 2013. “Liver Fat Content and Lipid Metabolism in Dairy Cows during Early Lactation and during a Mid-Lactation Feed Restriction.” J Dairy Sci 96(8). Elsevier: 5008–17. doi:10.3168/jds.2012-6245. 10.3168/jds.2012-6245 23746584

[CR11] Grummer RR, Hoffman PC, Luck ML, Bertics SJ. 1995. “Effect of Prepartum and Postpartum Dietary Energy on Growth and Lactation of Primiparous Cows.” J Dairy Sci 78(1). Elsevier: 172–80. doi:10.3168/jds.S0022-0302(95)76627-9. 10.3168/jds.S0022-0302(95)76627-9 7738253

[CR12] Herdt TH (2000). Ruminant Adaptation to Negative Energy Balance. Influences on the Etiology of Ketosis and Fatty Liver. Vet Clin North Am Food Anim Pract.

[CR13] Holtenius K, Agenäs S, Delavaud C, Chilliard Y (2003). Effects of Feeding Intensity during the Dry Period. 2. Metabolic and Hormonal Responses. J Dairy Sci.

[CR14] Huzzey JM, von Keyserlingk MA, Weary DM. 2005. “Changes in Feeding, Drinking, and Standing Behavior of Dairy Cows during the Transition Period.” J Dairy Sci 88(7). Elsevier: 2454–61. doi:10.3168/jds.S0022-0302(05)72923-4. 10.3168/jds.S0022-0302(05)72923-4 15956308

[CR15] Imhasly S, Naegeli H, Baumann S, von Bergen M, Luch A, Jungnickel H (2014). Metabolomic Biomarkers Correlating with Hepatic Lipidosis in Dairy Cows. BMC Vet Res.

[CR16] Ingvartsen VM (2006). “Feeding- and Management-Related Diseases in the Transition Cow.”. Anim Feed Sci Technol.

[CR17] Ingvartsen KL, Andersen JB (2000). Integration of Metabolism and Intake Regulation: A Review Focusing on Periparturient Animals. J Dairy Sci.

[CR18] Jacobs RL, van der Veen JN, Vance DE (2013). “Finding the Balance: The Role of S-Adenosylmethionine and Phosphatidylcholine Metabolism in Development of Nonalcoholic Fatty Liver Disease. Hepatology.

[CR19] Jorritsma R, Jorritsma H, Schukken YH, Bartlett PC, Wensing T, Wentink GH (2001). Prevalence and Indicators of Post Partum Fatty Infiltration of the Liver in Nine Commercial Dairy Herds in The Netherlands. Livestock Production Science.

[CR20] Kessler EC, Gross JJ, Bruckmaier RM, and Albrecht C. 2014. “Cholesterol Metabolism, Transport, and Hepatic Regulation in Dairy Cows during Transition and Early Lactation.” J Dairy Sci 97(9). Elsevier: 5481–90. doi:10.3168/jds.2014-7926. 10.3168/jds.2014-7926 24952770

[CR21] Matyash V, Liebisch G, Teymuras V, Kurzchalia AS, Schwudke D (2008). Lipid Extraction by Methyl-Tert-Butyl Ether for High-Throughput Lipidomics. J Lipid Res.

[CR22] McArt J, Nydam DV, Oetzel GR, Overton TR, Ospina PA. 2013. “Elevated Non-Esterified Fatty Acids and Β-Hydroxybutyrate and Their Association with Transition Dairy Cow Performance.” Veterinary Journal (London, England : 1997) 198(3). Elsevier Ltd: 560–70. doi:10.1016/j.tvjl.2013.08.011 10.1016/j.tvjl.2013.08.011 24054909

[CR23] Mølgaard L, Damgaard BM, Bjerre-Harpøth V, Herskin MS (2012). Effects of Percutaneous Needle Liver Biopsy on Dairy Cow Behaviour. Res Vet Sci.

[CR24] Moyes KM, Larsen T, Ingvartsen KL. 2013. “Generation of an Index for Physiological Imbalance and Its Use as a Predictor of Primary Disease in Dairy Cows during Early Lactation.” J Dairy Sci 96(4). Elsevier: 2161–70. doi:10.3168/jds.2012-5646. 10.3168/jds.2012-5646 23403197

[CR25] Ogiso H, Suzuki T, Taguchi R (2008). Development of a Reverse-Phase Liquid Chromatography Electrospray Ionization Mass Spectrometry Method for Lipidomics, Improving Detection of Phosphatidic Acid and Phosphatidylserine. Anal Biochem.

[CR26] Ospina PA, Nydam DV, Stokol T, Overton TR. 2010. “Associations of Elevated Nonesterified Fatty Acids and Beta-Hydroxybutyrate Concentrations with Early Lactation Reproductive Performance and Milk Production in Transition Dairy Cattle in the Northeastern United States.” J Dairy Sci 93(4). Elsevier: 1596–1603. doi:10.3168/jds.2009-2852. 10.3168/jds.2009-2852 20338437

[CR27] Pluskal T, Castillo S, Villar-Briones A, Oresic M (2010). MZmine 2: Modular Framework for Processing, Visualizing, and Analyzing Mass Spectrometry-Based Molecular Profile Data. BMC Bioinformatics.

[CR28] R_Core_Team (R Foundation for Statistical Computing). 2014. “R: A Language and Environment for Statistical Computing.” Vienna, Austria. http://www.r-project.org.

[CR29] Schmelzer K, Fahy A, Subramaniam S, Dennis EA. 2007. “The Lipid Maps Initiative in Lipidomics.” Methods Enzymol 432(07): 171–83. doi:10.1016/S0076-6879(07)32007-7 10.1016/S0076-6879(07)32007-7 17954217

[CR30] Sejersen H, Sørensen MT, Larsen T, Bendixen E, Ingvartsen KL. 2012. “Liver Protein Expression in Dairy Cows with High Liver Triglycerides in Early Lactation.” J Dairy Sci 95(5). Elsevier: 2409–21. doi:10.3168/jds.2011-4604. 10.3168/jds.2011-4604 22541469

[CR31] Sordillo LM, Raphael W (2013). Significance of Metabolic Stress, Lipid Mobilization, and Inflammation on Transition Cow Disorders. Vet Clin North Am Food Anim Pract.

[CR32] Van den Top AM, Van Tol A, Jansen H, Geelen MJ, Beynen AC. 2005. “Fatty Liver in Dairy Cows Post Partum Is Associated with Decreased Concentration of Plasma Triacylglycerols and Decreased Activity of Lipoprotein Lipase in Adipocytes.” J Dairy Res 72(2): 129–37. doi:10.1017/S0022029905000774. 10.1017/s0022029905000774 15909677

